# Development and Validation of Amplicon‐Based Protocol for Sequencing of Respiratory Syncytial Virus Genome

**DOI:** 10.1002/jmv.70571

**Published:** 2025-08-20

**Authors:** Guglielmo Ferrari, Great Romano, Antonino Maria Guglielmo Pitrolo, Fausto Baldanti, Antonio Piralla

**Affiliations:** ^1^ Microbiology and Virology Department Fondazione IRCCS Policlinico San Matteo Pavia Italy; ^2^ Department of Clinical, Surgical, Diagnostic and Pediatric Sciences University of Pavia Pavia Italy

**Keywords:** amplicon‐based protocol, molecular epidemiology, next‐generation sequencing, respiratory syncytial virus, whole‐genome sequencing

## Abstract

The most prevalent cause of severe respiratory infections in children is the human respiratory syncytial virus (RSV). The advent of next‐generation sequencing (NGS) has made it possible to incorporate this technology into pathogen monitoring and surveillance. Whole‐genome sequencing (WGS) of RSV has now become a relatively widely used method for tracking viral evolution. Here we report an improved high‐throughput RSV‐WGS assay performed directly on clinical samples that is suitable for short‐read sequencing platforms. A total of 100 RSV‐positive samples collected between November 2022 and March 2024 fulfilled the inclusion cycle quantification criteria and were randomly included in the validation process. The WGS protocol was designed to amplify three distinct amplicons to cover the entire RSV genome. The protocol described here can be successfully replicated in several instances (approximately 95%) in samples with a relatively low viral load, typically corresponding to cycle of quantification values of 27–32. The amplicon‐based protocol produced meaningful sequencing results in terms of median depth of coverage (more than 12000×) and median of mapped reads (> 1 × 10^6^ reads). The sequences that had passed the filters showed a coverage of at least 98% across the entire genome, with cycle quantification values of 32. Based on the obtained data resulting in an easy‐to‐perform protocol helpful for the molecular epidemiology surveillance of RSV.

## 1 Introduction

Human respiratory syncytial virus (RSV) is the leading cause of respiratory hospitalization of infants and is the second largest cause of lower respiratory infection mortality worldwide [1]. Infection rates typically rise in late autumn and early winter causing bronchiolitis in infants, common colds in adults and insidious respiratory illness in the elderly [1]. It is estimated that approximately 34 million new cases of lower respiratory tract infections (LRTIs) caused by RSV occur annually in children under the age of five [2]. Furthermore, 99% of the global childhood deaths caused by RSV infection occur in developing countries [2]. Among hospitalized patients, RSV has been estimated to cause 2.3% of deaths among neonates, 6.7% of deaths among infants beyond the neonatal age and, 1.6% of deaths among children between 1 and 4 years of age [3].

RSV is an enveloped virus belonging to the *Pneumoviridae* family. It is a negative‐sense, single‐stranded RNA virus comprising 11 proteins, encoded by a 15.2‐kb genome. The virus is classified into two major types: RSV‐A and RSV‐B. RSV classification is conventionally based on the G protein owing to its high degree of variability. However, other regions of the RSV genome, despite their reduced level of variability, are nevertheless also of relevance for surveillance purposes [4]. A recent revisions of RSV subtypes A and B classification was proposed with the objective of harmonising the criteria and nomenclature employed to describe these viruses [5]. This revision has been provided by the availability of an increasing number of full‐genome sequences and fully implemented in the Nextstrain platform (https://nextstrain.org/rsv) [6]. The advent of next‐generation sequencing (NGS) 20 years ago marked the commencement of a novel era in sequencing. This development has contributed to the integration of NGS into the surveillance of pathogens. In this context, the whole genome sequences (WGS) of RSV have become a straightforward procedure, supporting epidemiological surveillance to trace viral evolution [7, 8]. The World Health Organization (WHO) has also integrated RSV surveillance into its Global Influenza Surveillance and Response System to study the circulation and impact of RSV worldwide [9]. The development of WGS surveillance should ideally be available for multiple NGS platforms, an a greater benefit will be in the expansion of RSV surveillance to low and middle‐income countries where the burden of RSV is greatest [10]. Among the various NGS approaches, the PCR amplicon‐based NGS is a sensitive method for sequencing small genomes for population‐scale viral surveillance. In recent years, several protocols have been developed for the purpose of sequencing the entire RSV genome [11, 12, 13, 14, 15, 16, 17]. This approach has been demonstrated to be robust; however, the introduction of several primer pairs should be accompanied by a caveat regarding the variability of RSV during the time. An increase in the number of amplicons indicates the need to closely monitor the evolution of the virus to update the primers and avoid mismatches that could affect the sensitivity and quality of the WGS, as recently evaluated for SARS‐CoV‐2 [18]. In this respect, none of the studies describing the amplicon‐based approach have evaluated the quality of the primers against the sequence data set used for primer design or against RSV strains circulating in recent seasons [11, 12, 13, 14, 15, 16, 17]. In this context, the objectives of the present study were to: (i) develop a new PCR‐based next‐generation sequencing assay for WGS of RSV‐A and ‐B, (ii) assess the quality of primers using a phylo‐primer‐mismatch analysis and (iii) evaluate the performances of developed protocol and, (iv) sequence RSV strains circulating in northern Italy during the 2022–2024 winter seasons.

## 2 Methods

### 2.1 Primer Design Ad In‐Silico Evaluation

**Figure 1:**
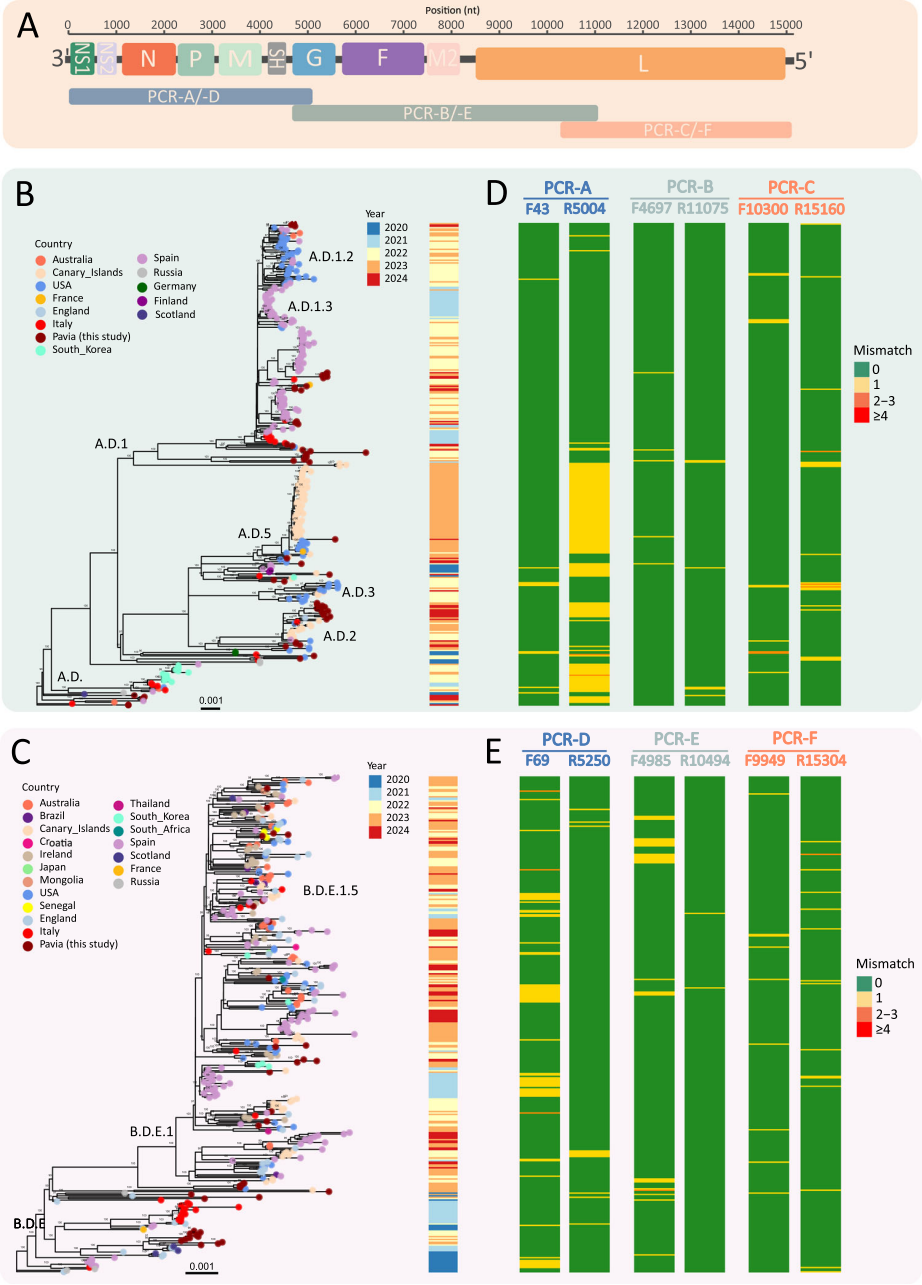
Phylogenetic and in silico analysis to assess the binding performance of primers for RSV‐A and RSV‐B. (A) The viral genome was divided into three segments with overlapping regions. Maximum likelihood phylogeny of RSV‐A (B) and RSV‐B (C) inferred from WGS. Phlyo‐primer mismatch graph to visualize of primer mismatches against the RSV‐A (D) and RSV‐B (E) phylogeny. Primer mismatches for each sequence were tabulated and overlaid on the tree. Sense primer, antisense primer, were evaluated in this order for each assay. Tip point colors represent the country of origin of RSV sequences including those originated from this study (in brown). Scale bars indicate 0.001 nucleotide substitution per site.

A total of 709 complete genome sequences of RSV‐A and RSV‐B circulated in the 2020–2024 period and corresponding to genotypes AD.1‐AD.5 for RSV‐A and B.D, BD.4 and B.D.E.1‐4 (https://nextstrain.org/rsv/a/genome/6y) were retrieved from an online repository (Nextstrain, accessed on 01 April 2025) and aligned using MAFFT v7.525 [19]. Primers for RT‐PCR were designed based on conserved regions of RSV‐A and ‐B in silico PCR using FastPCR software [20]. The specificity of these primers was further verified by BLAST to ensure that they were specific for RSV. The design process resulted in three pairs of primer designed to amplify three distinct amplicons for RSV‐A and RSV‐B (Table 1 and Figure 1A). All sequences were then mapped to reference genomes for RSV‐A and RSV‐B, and the most frequently mapping primers in the overlap region between two contiguous segments were selected. Primer efficiencies and the theoretical annealing temperature were evaluated using the FastPCR software [20]. Primer sequences were mapped against the alignment to analyse the number of mismatches per strain (phylo‐primer‐missmacth analysis). The mismatches were tabulated for visualization and overlayed with the phylogenetic tree as previously reported by Giardina et al. [21] using an in‐house script developed in the R programming language (https://www.r-project.org/) [22].

**Table 1:** RT‐PCR schemes and primers list developed for WGS.

		Primer	Primer sequence	Amplicon length
RSV‐A	PCR A	F43	atggggcaaataagaatttgataagtacca	4961 bp
		R5004	cggacagattggagaagctgattcca	
	PCR B	F4697	aacatgtccaaaaccaaggaccaacgcac	6378 bp
		R11075	tctgcttgagcatgagtttgaccttcca	
	PCR C	F10300	atgtggtatcattgacaggcaaagaa	4860 bp
		R15160	tgaatacaatgttagtgtgtagc	
RSV‐B	PCR D	F69	atgcgtactacaaacttgcacactcg	5181 bp
		R5250	gtggcaacaatcaactctgcaaatcc	
	PCR E	F4985	caagtctcaccagaaagggttagcccatcc	5509 bp
		R10494	gcctaaacatacctggttgcatagca	
	PCR F	F9949	tagcctaagtacgttgagaggtgc	5355 bp
		R15304	actaatgtctcgttgtgttgtaaatgca	

### 2.2 Molecular Screening for RSV

A total of 2761 respiratory samples were collected between November 2022 and March 2024 and tested with a panel of real‐time RT‐PCRs for respiratory viruses at Microbiology and Virology Department, Fondazione IRCCS Policlinico San Matteo, Pavia. Viral genome extraction was performed on the QIAsymphony platform using the “Virus Pathogens DSP Midi Kit” (Qiagen, Heidelberg, Germany), starting from a volume of 400 μL with a final elution of 60 μL. Real‐time RT‐PCRs were performed to detect and quantify the following viruses: influenza virus A (IVA), influenza virus B (IVB), human rhinoviruses (HRVs), enteroviruses (EVs), respiratory syncytial virus (RSV), human coronaviruses (hCoV−OC43, −229E, ‐NL63, and ‐HKU1), as previously described [23, 24]. Typing of RSV‐positive samples was performed by real‐time RT‐PCR specific for RSV‐A and RSV‐B using AgPath‐ID One‐Step RT‐PCR enzyme (Applied Biosystems) as previously reported [24]. Results of RSV‐specific real‐time RT‐PCR [24] were reported as cycle of quantification (Cq) values which are inversely proportional to viral load. A Cq ≥ 40 was used as the negative cut‐off. To validate the WGS protocol, a series of RSV‐positive samples with Cq ≤ 32 (corresponding to nearly ≥ 10^3.5^ RNA copies/mL quantified using RSV‐specific real‐time RT‐PCR and standards curve [24]) and a minimum of residual sample volume of 500 µL were included in the validation analysis.

### 2.3 Whole‐Genome Amplification and Sequencing

SuperScript IV One‐Step RT‐PCR System enzyme (ThermoFisher Scientific, Waltham, MA, USA) was used for RSV whole genome amplification with three separate RT‐PCR reactions. Briefly, 50 µL reaction containing 10 µL total RNA, 0.5 µL of SuperScript IV RT Mix (ThermoFisher Scientific, Waltham, MA, USA), 2.5 µL of each primer (10 µM) at 0.5 µM final concentration, 25 µL PlatinumTM SuperFi RT‐PCR Master Mix (ThermoFisher Scientific, Waltham, MA, USA) and 9.5 µL of nuclease‐free water. The following cycling conditions were used: 25 min at 55°C for reverse transcription, 2 min at 98°C followed by 40 cycles of 98°C for 10 s, 60°C for 10 s and 72°C for 3 min and 30 s, with a final extension of 72°C for 5 min. The presence of amplicons was verified by electrophoretic gel (1% Agarose; see Supporting Information S1: Figure 1). Amplicons were purified using AMPure XP beads and eluted in 50 µL of 1× TE buffer and quantified using the Qubit dsDNA BR Assay Kit and QubitTM 4 Fluorometer (ThermoFisher Scientific, Waltham, MA, USA). Equal amounts of RT‐PCR products from three amplicons were pooled together for downstream NGS library preparation and sequencing. Genomic libraries were prepared using the Nextera XT Library Preparation Kit (Illumina, San Diego, CA, USA) according to the manufacturer's instructions. Libraries were quantified using Qubit 1× dsDNA HS Assay kit, normalized to the same concentration, and then pooled together. The pooled libraries were quantified by the Qubit dsDNA High Sensitivity assay (ThermoFisher Scientific, Waltham, MA, USA). The sizes of pooled libraries were measured with D1000 ScreenTape and reagents on a TapeStation device (Agilent Technologies, Santa Clara, CA, USA). Based on the results from the Qubit and TapeStation measurements, pooled libraries were diluted to 10 pM and sequenced on an Illumina Dx‐MiSeq sequencer using paired‐end runs with 300 cycles (Illumina, San Diego, CA, USA). CZ ID bioinformatics tool was used to perform read quality control, adapter trimming and to assemble viral consensus genomes [25].

### 2.4 Validation Criteria

Sequenced samples were considered of “good quality” when they displayed ≥ 98% genome coverage at a minimum read depth of 30x unique reads per position. Samples with genome coverage below 98% and 30× read depth were classified as failed.

### 2.5 Phylogenetic Analysis of RSV Strains

The alignment was performed with the data set aligned with MAFFT v7.525 [19], visualized with MEGA11 [26]. A Maximum Likelihood (ML) phylogenetic tree of the data set was constructed using IQ‐TREE multicore version 2.3.3 [27] under a nucleotide substitution model chosen according to BIC score (i.e., Bayesian Information Criterion), as it was the best‐fitting one selected by ModelFinder and presented in Figure 1B,C [28]. The robustness of branches was evaluated using the Shimodaira–Hasegawa approximate likelihood‐ratio test (SH‐aLRT) [29] and ultrafast bootstrap approximation tests [30]. The phylogenetic tree was visualized using ggtree package [31] and in‐house R script [22]. GISAID accession number of sequences originated in the study are from EPI_ISL_18329452 to EPI_ISL_19742937 for RSV‐A and EPI_ISL_18329463 to EPI_ISL_18329491 for RSV‐B.

### 2.6 Statistical Analysis and Ethical Issue

Comparisons between two groups were performed using the two‐tailed *t*‐test. *p* values less than 0.05 were considered as statistically significant. R software and packages were used for making graphs and curve‐fitting analyses. All the statistical analyses were performed using GraphPad Prism software version 8.3 (GraphPad Software, San Diego, CA). The study HS‐2024‐001 was approved by the Regional ethical committee (CET Lombardia 1).

## 3 Results

### 3.1 PCR Primer Design In Silico

To validate the PCR primers used in this study, we performed an in‐silico analysis to evaluate the performances in terms of degree of primers mismatch. These mismatches per primer corresponding to of the position in the tree for RSV‐A and RSV‐B sequences included in the data set were showed in Figure 1D,E. For each sequence of the data set aligned with the primer, the number of mismatches was count and reported as categorical variable (i.e., 0, 1, 2‐3, ≥ 4 mismatches) in the heatmap (Figure 1D,E). Overall, all the primers had a good performance, mainly highlighted by the green colour where no mismatches were detected. In detail, among the forward primers for RSV‐A, F4697 had the best performance with 351 out of 356 (98.6%) sequences without mismatches followed by F43 (347/356; 97.5%) and F10300 (345/356; 96.9%) (Figure 1D and Supporting Information S1: Table S1). Regarding reverse primers, R11075 had the best performance with 350 out of 356 (98.3%) sequences without mismatches, followed by R15160 (337/356; 94.7%) and R5004 (233/356; 65.4%) (Supporting Information S1: Table S1). In this latter primer, one mismatch was detected in 121/356 (34.0%) sequences. Multiple mismatches (2 or more) were scantly observed and only for a few RSV‐A strains (less than 1.0% of total) (Figure 1D and Supporting Information S1: Table S1). Among the forward primers for RSV‐B, F9949 had the best performance with 344 out of 353 (96.6%) sequences without mismatches sequences followed by F4985 (325/353; 91.3%) and F69 (295/353; 82.9%) (Figure 1E). Regarding reverse primers, R10494 had the best performance with 351 out of 353 (98.6%) sequences without mismatches, followed by R5250 (342/353; 96.1%) and R15304 (338/353; 94.9%) (Supporting Information S1: Table S1). Multiple mismatches (2 or more) were scantly observed and only for few RSV‐B strains (less than 1.0% of total).

### 3.2 Amplicon‐Based Protocol Validation

To validate our WGS protocol, we evaluated assay performance by directly sequencing clinical samples. A total of 303/2761 (10.9%) samples were RSV‐positive from November 2022 to March 2024. Of these, 100 RSV‐positive samples fulfilling the inclusion criteria were randomly selected for the validation process. Real‐time RT‐PCR typing revealed 58 RSV‐A, 41 RSV‐B and 1 co‐infection RSV‐A and RSV‐B (excluded from the analysis), with a Cq value between 13 and 32.

In 94/99 (94.9%) RSV‐positive samples (55 RSV‐A and 39 RSV‐B), the three amplicon bands were clearly detectable in gel electrophoresis that reflects that the targeted PCR product was correctly amplified (Supporting Information S1: Figure S1). For these samples, the median of genome coverage obtained was 99.0% (IQR 98.9%–99.1%) for RSV‐A and 99.7% (IQR 99.5%–99.8%) for RSV‐B. In 5/99 (5.1%) samples (3 RSV‐A and 2 RSV‐B) not all three amplicons were detectable in gel electrophoresis and resulted in a genome coverage ranging from 68.2% to 96.2%. Partial genome sequencing does not seem to correlate with low viral load, but rather with technical failure, as the Cq values of these samples ranged from 19 to 28 (19, 23, 23, 24, 28). However, a depth of coverage greater than 1000× were obtained for complete sequences of the G and F genes.

**Figure 2:**
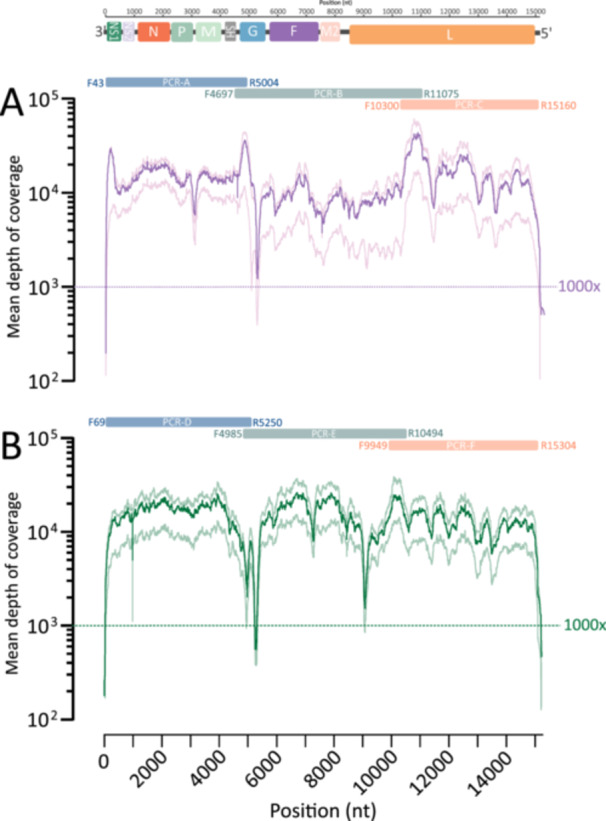
Genome coverage map for (A) RSV‐A and (B) RSV‐B. The mean coverage for each site is reported in violet and green with IQR*;* 25*%* and 75% in softened colour.

**Figure 3:**
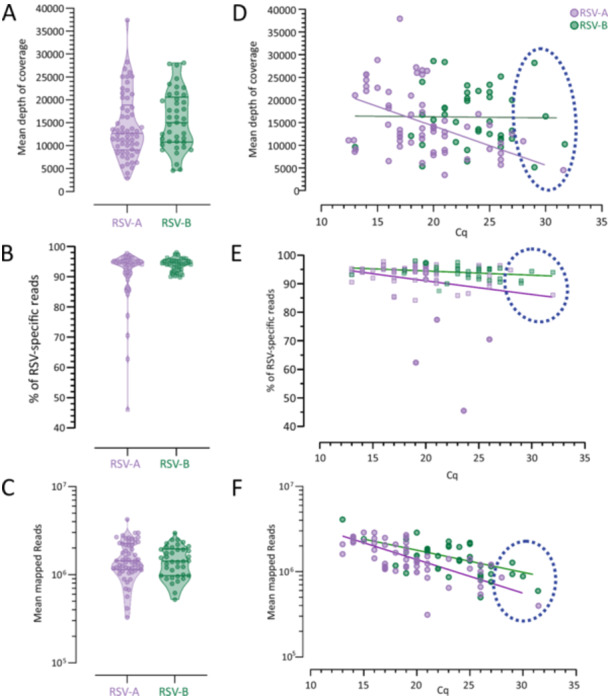
Violin plots showing (A) the mean depth (B) the percentage of RSV‐specific reads and (C) the mean mapped reads for RSV‐A in violet and RSV‐B in green. Correlation between Cq and (D) mean depth of coverage (E) the percentage of RSV‐specific reads and (F) mean of mapped reads for RSV‐A in violet and RSV‐B in green. Samples with Cq values ranging from 29 to 32 are indicated by a dot blue circle.

Overall, in the 94 the nearly full‐genome sequences, the median depth of coverage was 12,685× (IQR 9081×–18,874×) for RSV‐A and 15068× (IQR 10810×–20598×) RSV‐B (Figures 2 and 3A). RSV‐specific reads accounted for a median of 94.5% of the total reads (IQR 91.0%–95.2%) for RSV‐A and 94.3% of the total reads (IQR 92.3%–95.4%) for RSV‐B (Figure 3B). In addition, the median of mapped reads was 1.44 × 10^6^ (IQR 1.14 × 10^6^–2.27 × 10^6^) and 1.42 × 10^6^ (IQR 9.75 × 10^5^–1.95 × 10^6^) for RSV‐A and RSV‐B, respectively (Figure 3C). Of note, in samples with the lower viral load (Cq values ranging from 29 to 32), the number of mapped reads against RSV was always higher than 5 × 10^5^.

The correlation analysis reveals a significant linear relationship between mean depth coverage or mapped reads and Cq value for RSV‐A and only for mapped reads in RSV‐B strains (Figure 3D,F).

Furthermore, no difference was observed in the % of RSV‐specific reads obtained according to Cq demonstrating a high specificity of reads obtained after sequencing process (Figure 3E). In detail, Spearman's rank correlation coefficient between Cq and mean depth coverage was −0.50 (95% CI, −0.68 to −0.28) for RSV‐A and −0.05 (95% IC, −0.36 to 0.26) for RSV‐B. Spearman's rank correlation coefficient between Cq and mean of mapped reads was −0.71 for RSV‐A (95% CI, −0.82 to −0.54) and −0.59 for RSV‐B (95% CI −0.76 to −0.34). Furthermore, we compared the % of ambiguous bases and Ns or failed bases with the informative nucleotides. The ambiguous bases metric specifies the number of non‐C, T, G, A nucleotides in the consensus genome; if multiple sequencing reads support more than one nucleotide at a given site, those sites will be designated with an IUPAC ambiguity code. The consensus genome pipeline only calls nucleotides that are detected at least at 75% frequency. The ideal number of ambiguous bases to achieve high genome quality is below 10, corresponding to a percentage of 0.06. Ninety genomes (90.9%) contain < 0.5% of Ns or failed base calls and contain < 0.06% of ambiguous bases.

### 3.3 Phylogenetic Analysis of RSV Strains

Phylogenetic analyses were performed to assess the relationships amongst 2023/2024 sequences collected from Pavia territory globally with globally strains deposited in GISAID and collected in the period 2020–2024 (Figure 1B,C). The results indicate that the RSV‐A sequences are primarily arranged as multiple single insertions, except for the formation of two minor clades (Figure 1B). The upper clade, comprising primarily strains from 2022, exhibits a closer relationship with Spanish strains. The lower clade, which is composed primarily of strains from 2024, exhibits a closer phylogenetic relationship with strains from the Canary Islands. The latter are predominantly dated to 2023. This suggests an evolutionary correlation that may have led to the spread of RSV‐A strains in our territory in 2024 (Figure 1B). In the case of RSV‐B sequences, the phylogenetic tree reveals the formation of a single cluster comprising strains primarily from 2022 to 2023. This cluster can be traced back to a new viral introduction to our country (Figure 1C).

## 4 Discussion

Understanding the epidemiology and evolution of RSV is critical for the development of effective public health strategies, including vaccines and antiviral treatments [32]. WGS has recently emerged as a powerful tool for investigating the genetic diversity, transmission dynamics and evolutionary patterns of RSV [32]. This sequencing activity has undoubtedly benefited from the technical expertise and infrastructure acquired worldwide during the COVID‐19 pandemic. Recent technical improvements to WGS workflows include the use of amplicon‐based NGS sequencing also for RSV [11, 12, 13, 14, 15, 16, 17]. All these protocols are based on the amplicon approach and designed to use several amplicons ranging from 4 to 25. The present study proposes a new amplicon‐based WGS protocol that aims to minimise primer pair usage with only three primer pairs. Our protocol was validated using RSV strains circulating in the most recent RSV seasons. Amplicon‐based WGS protocols is available for several sequencing platforms (usually Illumina or Oxford Nanopore) to sequence amplicons generated by RT‐PCR, using primers targeted to conserved regions of the RSV‐A and RSV‐B genomes. An alternative strategy is to use an enrichment NGS approach using target‐capture probes that select nucleic acid of specific pathogen. However, although it has a high specificity of binding to target sequences and requires fewer PCR cycles (compared to the amplification protocol), it is already expensive and requires more technical expertise than the amplification protocol. Amplicon approach is highly versatile and can be an efficient method for WGS of pathogens with small to medium‐sized genomes. As previously outlined by Kongen et al. [33], design of overlapping PCRs can facilitate the enrichment of the viral genome, thereby enhancing the quality of the sequence and reducing costs [33]. However, in cases where primer mismatches occur, amplicon sequencing may result in the formation of sequencing gaps [19]. Previously reported amplicon‐based WGS protocols for RSV did not address this issue, considering only the evaluation of performances [11, 12, 13, 14, 15, 16, 17]. In our study, in‐silico analyses of the primers indicate a robust binding performance relative to the local and global RSV strains included in the analysis. In addition, RSV variability occurred in strains circulating from 2020 to 2024 does not appear to have a detrimental effect on primer binding performance.

Overall, our protocol allows at least 95% of RSV‐positive samples with viral load ≥ 10^3.5^ RNA copies/mL (Cq ≤ 32) to be sequenced with genome coverage greater than 98%. Our findings are in keeping with those published by Talts et al. with a success rate of sequencing > 90% in samples with Cq < 31 [14]. In other reports, comparable genome coverage performance was achieved only with samples containing a higher amount of RNA Cq < 25 [15] or Cq < 23 [11]. Our amplicon‐based protocol showed excellent sequencing parameters such as a median depth of coverage higher than 12000× and more than 1.6 × 10^6^ mapped reads for RSV‐A and RSV‐B including a rate of RSV‐specific reads of more than 94% of the total obtained reads. We have also observed few samples the partial presence of amplified PCR products that are not detectable on an electrophoretic gel. However, this does not necessarily mean that the amplification has failed. This finding is in keeping with those observed by Dong et al. [12], indicating that WGS can also function effectively with weak PCR fragments that are not discernible on a TapeStation system or electrophoretic gels. The resulting sequences have a coverage between 68.2% and 96.2% across the genome. Of these, the five partial genome sequences (F and G genes complete) showed sequencing depth greater than 1000× also useful to monitor viral evolution on surface glycoprotein. Our protocol was developed without the inclusion of DNAse treatment of samples before amplification as previously reported to improve sequencing performance [12, 34]. It has been suggested that this may be an effective method for processing samples with medium to high Cq values (Cq 23–28) [12, 34]. Based on the good performance of our protocol, which demonstrated high sequencing yields in samples with Cq 26 to 32, we believe that DNAse treatment could be avoided using the protocol presented here. Finally, though a correlation between Cq and partial genomes has not been evidenced, an important aspect to consider is the quality of the RNA used, especially when, as in our protocol, very long amplicons (> 4500 bp) are used.

WGS has improved the genotyping assignment for RSV‐A and RSV‐B and a more standardized classification of emerging RSV strains has been suggested by Chen et al. [5]. Furthermore, despite the increasing availability of sequencing data, its processing and interpretation require scientists trained in bioinformatics. In this regard, Dosbaa et al. presented an accessible software and automated pipeline for WGS analysis of RSV sequences to facilitate genomic analyses by individuals without bioinformatics training [35]. In the present study, an easy‐to‐use method for the reconstruction of the whole RSV genome has been used. This method employs a web‐based bioinformatics platform (https://czid.org/) with a user‐friendly interface. The platform is also useful for metagenomic analysis of shotgun sequences.

The introduction of authorised vaccines and monoclonal antibodies for RSV should be supported by an increased global focus on virus epidemiology. Furthermore, greater attention should be paid to the evolution of the virus, with particular attention to the evolution of antigenic sites in the F protein, which may reduce the efficacy of vaccines or enable escape from therapeutics [36]. WGS not only provides a snapshot of the current state of RSV genetic diversity, but it also offers predictive power for anticipating future evolutionary trends. By analysing genetic variation over time, scientists can estimate the rate of mutation and predict how RSV might evolve in response to new vaccines, antiviral drugs, or changes in population immunity [37]. For instance, Rios‐Guzman et al. [38] demonstrated how genomic surveillance of RSV could be used to predict shifts in viral subtypes over time.

This study has several limitations. Firstly, a performance comparison with previously published sequencing methods is lacking, which restricts the contextual evaluation of its effectiveness. Secondly, the integrity of the RNA was not assessed before PCR amplification, which may have resulted in poor‐quality sequences, even in samples with an adequate viral load.

In conclusion, WGS has changed the study of RSV, offering significant advantages in both epidemiology and the study of viral evolution. The present protocol has been developed for the implementation of WGS with the objective of facilitating the monitoring of RSV dynamics and evolution in the era of vaccine. Furthermore, the utilisation of a freely accessible web‐based bioinformatic platform enables individuals to perform reproducible bioinformatic analyses via a graphical user interface.

## Author Contributions

Conceptualization: Antonio Piralla. Methodology: Guglielmo Ferrari, Great Romano, Antonino Maria Guglielmo Pitrolo. Validation: Guglielmo Ferrari, Great Romano. Writing – original draft preparation: Guglielmo Ferrari, Great Romano. Writing – review and editing: Antonio Piralla. Supervision: Fausto Baldanti.

## Ethics Statement

The study HS‐2024‐001 was approved by the Regional ethical committee (CET Lombardia 1).

## Conflicts of Interest

The authors declare no conflicts of interest.

## Supporting information


**Supplementary Figure 1:** Gel electrophoresis picture of amplicons for RSV‐A and RSV‐B strains. Molecular wight VIII was used as control (Roche cat. No. 11336045001).
**Table S1:** Summary of the number of mismatches observed for each primer employed for the amplification of the complete genome (n=709).

## Data Availability

The data that support the findings of this study are available from the corresponding author upon reasonable request.
