# Supplementary material for: Development and Validation of Amplicon‐Based Protocol for Sequencing of Respiratory Syncytial Virus Genome
Source: J Med Virol. 2025 Aug 20;97(8):e70571. doi: 10.1002/jmv.70571 (PMC12365941; doi:10.1002/jmv.70571)

**Table S1.** Summary of the number of mismatches observed for each primer employed for the amplification of the complete genome (n=709).

| RSV subtype (strain analyzed) | Primer | N° mismatches | | | |
| --- | --- | --- | --- | --- | --- |
|  |  | 0 | 1 | 2-3 | ≥4 |
| RSV-A (356) | F43 | 347 (97.5%) | 9 (12.5%) | 0 | 0 |
|  | R5004 | 233 (65.4%) | 121 (34.0%) | 2 (0.6%) | 0 |
|  | F4697 | 351 (98.6%) | 5 (1.4%) | 0 | 0 |
|  | R11075 | 350 (98.3%) | 6 (1.7%) | 0 | 0 |
|  | F10300 | 345 (96.9%) | 9 (2.5%) | 2 (0.6%) | 0 |
|  | R15160 | 337 (94.7%) | 16 (4.5%) | 3 (0.8%) | 0 |
| RSV-B (353) | F69 | 295 (82.9%) | 55 (15.4%) | 3 (0.8%) | 0 |
|  | R5250 | 342 (96.1%) | 11 (3.1%) | 0 | 0 |
|  | F4985 | 325 (91.3%) | 27 (7.6%) | 1 (0.3%) | 0 |
|  | R10494 | 351 (98.6%) | 2 (0.6%) | 0 | 0 |
|  | F9949 | 344 (96.6%) | 9 (2.5%) | 0 | 0 |
|  | R15304 | 338 (94.9%) | 14 (3.9%) | 1 (0.3%) | 0 |

**GISAID Accession Numbers**

RSV-A

EPI_ISL_19742902, EPI_ISL_19742924, EPI_ISL_19742903, EPI_ISL_19742925, EPI_ISL_19742904, EPI_ISL_19742926,

EPI_ISL_19742905, EPI_ISL_19742927, EPI_ISL_19742920, EPI_ISL_19742921, EPI_ISL_19742900, EPI_ISL_19742922, EPI_ISL_19742901, EPI_ISL_19742923, EPI_ISL_19742906, EPI_ISL_19742928, EPI_ISL_19742907, EPI_ISL_19742929, EPI_ISL_19742908, EPI_ISL_19742909, EPI_ISL_19742913, EPI_ISL_19742935, EPI_ISL_19742914, EPI_ISL_19742936, EPI_ISL_19742915, EPI_ISL_19742937, EPI_ISL_19742916, EPI_ISL_19742898, EPI_ISL_19742931, EPI_ISL_19742899, EPI_ISL_19742910, EPI_ISL_19742932, EPI_ISL_19742911, EPI_ISL_19742933, EPI_ISL_19742912, EPI_ISL_19742934, EPI_ISL_19742894, EPI_ISL_19742895, EPI_ISL_19742896, EPI_ISL_19742897, EPI_ISL_19742930, EPI_ISL_19742891, EPI_ISL_19742892, EPI_ISL_19742893, EPI_ISL_19742917, EPI_ISL_19742918, EPI_ISL_19742919, EPI_ISL_18329458, EPI_ISL_18329459, EPI_ISL_18329456, EPI_ISL_18329457, EPI_ISL_18329460, EPI_ISL_18329454, EPI_ISL_18329455, EPI_ISL_18329452

RSV-B

EPI_ISL_19742968, EPI_ISL_19742979, EPI_ISL_19742969, EPI_ISL_19742975, EPI_ISL_19742976, EPI_ISL_19742977, EPI_ISL_19742978, EPI_ISL_19742971, EPI_ISL_19742972, EPI_ISL_19742973, EPI_ISL_19742974, EPI_ISL_19742980, EPI_ISL_19742970, EPI_ISL_18329463, EPI_ISL_18329464, EPI_ISL_18329465, EPI_ISL_18329466, EPI_ISL_18329467, EPI_ISL_18329468, EPI_ISL_18329469, EPI_ISL_18329470, EPI_ISL_18329471, EPI_ISL_18329473, EPI_ISL_18329474, EPI_ISL_18329475, EPI_ISL_18329478, EPI_ISL_18329479, EPI_ISL_18329480, EPI_ISL_18329481, EPI_ISL_18329482, EPI_ISL_18329483, EPI_ISL_18329484, EPI_ISL_18329485, EPI_ISL_18329486, EPI_ISL_18329487, EPI_ISL_18329488, EPI_ISL_18329489, EPI_ISL_18329490, EPI_ISL_18329491

**Supplementary Figure 1.** Gel electrophoresis picture of amplicons for RSV-A and RSV-B strains. Molecular wight VIII was used as control (Roche cat. No. 11336045001).


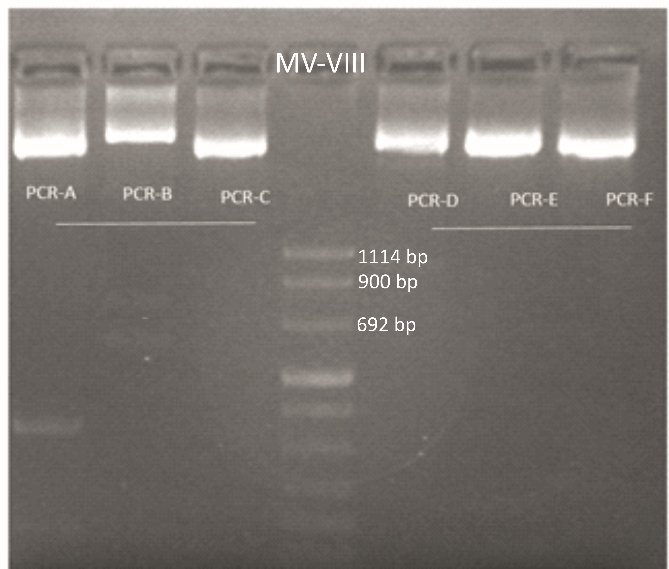

Supplement: Supplementary file 1 — Supplementary Figure 1: Gel electrophoresis picture of amplicons for RSV‐A and RSV‐B strains. Molecular wight VIII was used as control (Roche cat. No. 11336045001). Table S1: Summary of the number of mismatches observed for each primer employed for the amplification of the complete genome (n=709). [file JMV-97-e70571-s001.docx]
